# As Strong as We Are United: Effects of Intrapersonal and Interpersonal Emotion Regulation on Quality of Life in Women With Breast Cancer

**DOI:** 10.3389/fpsyg.2021.661496

**Published:** 2021-09-24

**Authors:** Rita Moura, Cristina Camilo, Sílvia Luís

**Affiliations:** ^1^CIS-IUL, Iscte - Instituto Universitário de Lisboa, Lisbon, Portugal; ^2^Instituto Superior de Ciências Sociais e Políticas, Centro de Administração e de Políticas Públicas, Universidade de Lisboa, Lisbon, Portugal; ^3^Escola de Psicologia e Ciências da Vida, HEI-Lab, Universidade Lusófona de Humanidades e Tecnologias de Lisboa, Lisbon, Portugal

**Keywords:** breast cancer, emotion regulation, emotional sharing, negative emotions, wellbeing, quality of life, adaptation to illness

## Abstract

Women diagnosed with breast cancer often experience unpleasant emotions, resulting in higher levels of emotional burden and decreased levels of wellbeing and quality of life. The present correlational and cross-sectional study aims to compare the implementation of two regulatory levels, intrapersonal and interpersonal (as social sharing of emotions), and two types of strategies, antecedent-focused and response-focused, and explore their impact on breast cancer patients’ perception of quality of life. Sixty-eight women previously diagnosed with the disease participated in this study, with a mean age of 63years old (*SD*=11.58). Data were collected through a self-report questionnaire to assess emotional experience, intrapersonal regulation, social sharing of emotions, and breast cancer-related wellbeing and quality of life. Data yielded that most of the participants regulated their negative emotions within social interactions and made more use of antecedent-focused strategies to cognitively reformulate the emotional episode. Social and family wellbeing were positively associated with antecedent-focused strategies, as well as intrapersonal and interpersonal regulatory levels. Moreover, the occurrence of sharing episodes and social interactions played an important and beneficial role on patients’ perceived quality of life. These findings reinforce the importance of promoting an adaptive intrapersonal regulation among breast cancer patients. Results also suggest that social sharing of emotions is an efficient process to help them to better cope with the psychological and emotional burden of the disease, thus positively influencing the way they perceive their social and family wellbeing, as well as their quality of life.

## Introduction

Breast cancer is the most common type of malignant tumor among women, with 2.1 million cases worldwide, although mortality numbers have been declining in recent years (Global Cancer Observatory, 2018). This phenomenon can be explained by the increase in early diagnosis and treatment effectiveness, leading to higher survival rates (American Cancer Society, 2020). The diagnosis, treatment, and following remission phases often result in disruptive and unpleasant experiences that threaten patients’ mental health and increase their emotional vulnerability (Ng et al., 2017). Even though patients might experience pleasant emotions (e.g., a sense of personal growth) that benefit their perception of wellbeing and quality of life, they are often more vulnerable to the wide range of persistent negative emotions they feel due to the disease, with anxiety and depression being the most frequent psychological reactions associated with breast cancer (Maass et al., 2015). Other frequent emotional responses are fear (e.g., of dying, suffering, or recurrence), anger, uncertainty (e.g., about the future), shock, despair, frustration, and more rarely guilt. Once emotional vulnerability becomes acute, it can increase psychological distress and disease severity, significantly impairing treatment outcomes, recovery time, and adaptation to illness, even after being cancer free (Brandão et al., 2017a; Ng et al., 2017). Hence, an adaptative regulation of such unpleasant emotions is crucial to reach emotional stability and ease women’s overall experience with breast cancer.

When emotions are harmful, whether because they are the wrong type, intensity, duration, or frequency for a certain situation, patients can—and often try to—regulate their emotions and change the emotion trajectory (Gross, 2015), manipulating which emotions are felt, when, and how they are experienced and expressed throughout the emotional event (Gross, 1998a). Emotion regulation episodes can occur on an intrapersonal or interpersonal level, with different associated psychosocial outcomes (Nyklíček et al., 2011). Research has been mostly focused on intrapersonal regulatory processes, even though recent studies highlight that regulatory episodes often occur in social contexts (Barthel et al., 2018). In fact, it is common for individuals to influence one another’s emotions and co-regulate them, determining which strategies and outcomes arise from the social regulatory episode (Marroquín et al., 2017). Intrapersonal regulation, particularly, refers to strategies that people use to deal with their emotional experiences by themselves (Gross, 2015), while interpersonal regulation involves the presence of others to regulate one’s emotions with or through them (Barthel et al., 2018). As a result, the effectiveness of the intrapersonal process is strongly and exclusively linked to an individual’s inner capacity to regulate their emotions alone. In interpersonal processes, other individuals’ skills are also highly relevant to the success of the regulation, which can be most helpful when individuals do not know how to adequately reduce their unpleasant emotions and rely on others to better select effective regulatory strategies (Levy-Gigi and Shamay-Tsoory, 2017; Marroquín et al., 2017). Both regulatory levels exist on a continuum without a clear delimitation and can be used simultaneously or interchangeably during a single regulatory episode (Williams et al., 2018).

On the intrapersonal level, the process model of emotion regulation (Gross, 1998a) is the most widely used conceptual framework to explain the emotional dynamics that influence the way individuals feel, think, and act, both immediately after the emotional event and over time (Gross, 2015). This model builds on the steps that occur in the process of emotion generation, deeming each step as a potential target for regulation. Regulatory strategies are grouped in five families, namely, situation selection, situation modification, attention deployment, cognitive change, and response modulation. According to the model, each family can be distinguished between antecedent-focused and response-focused, based on their primary impact during the regulation process. Antecedent-focused strategies act before the emotional response is fully developed and alter the subsequent emotion trajectory (the first four families fall into this category), whereas response-focused strategies act after the emotional response has already begun and seek to modify external aspects, such as behavioral expression (the last family falls into this category; Gross, 1998b). Cognitive reappraisal, an antecedent-focused strategy (part of the cognitive change family), and emotional suppression, a response-focused strategy (part of the response modulation family), are two well-researched examples of intrapersonal regulation processes, frequently used to reduce the impact of negative emotions. Through reappraisal, individuals think about the situation from a different perspective as an attempt to change the emotional response (e.g., thinking that the disease can potentially be a positive personal growth experience), while suppression inhibits any verbal or non-verbal expression related to the emotion (e.g., trying to hide the emotional impact of going through another round of chemotherapy from their loved ones; Gross, 2015). Generally, an attempt to reappraise the emotional event is considered to be more effective than suppressing it (Webb et al., 2012), successfully redirecting to neutral or positive feelings (McRae, 2016). Thus, it is not uncommon for antecedent-focused strategies to be the focus of emotion regulation efforts (Gross, 2015). Contrarily, suppression involves a continuous and repetitive effort to deal with the lingering and unresolved emotion (Webb et al., 2012), which might be counterproductive, intensifying negative emotions or even repressing positive ones (Gross and John, 2003).

On the interpersonal level, social sharing of emotions is one of the most frequent responses to an emotional event, considering that, when people experience an emotion, they tend to feel a pressing need to talk about it, with 80 to 95% of the episodes being socially shared (Brans et al., 2013). During these social encounters, people openly talk about the circumstances and emotions associated with the event (Rimé, 2009). The two-mode model (Rimé, 2009) states that even though verbalization is beneficial, it is not enough to effectively deal with an unpleasant emotional event. It is also necessary to take into consideration the way people share their emotions. This model differentiates between two sharing modes that decrease the impact of a negative emotion: socio-affective sharing and cognitive sharing. Socio-affective sharing involves a listener that provides a supportive response based on comfort, validation, and empathy. For instance, letting them know that it is normal to feel upset for being diagnosed with breast cancer. This mode is usually more effective during the initial phase, leading to a temporary state of emotional relief. Conversely, cognitive sharing involves a listener that stimulates the other person to work toward reformulating or reassessing the meaning of the emotional event, considerably reducing its unpleasant impact. For example, helping one understand they are coping in the best possible way, given the circumstances. The premise of the model is that, in order to achieve a positive and prolonged emotional recovery, both modes need to be implemented during the sharing episode. Thus, individuals not only feel supported by others, but also actively resolve the emotional stressor associated with the negative event (Brans et al., 2013). For the purposes of the study, cognitive sharing is considered to have a focus on the antecedents of the emotion (i.e., thoughts), while socio-affective sharing focuses on the emotional response (i.e., emotional expression) to allow the comparison between intrapersonal and interpersonal regulation models.

Research demonstrates that both regulatory levels have been used among breast cancer patients. At the intrapersonal level, the use of suppression is linked to worse mental health outcomes (e.g., negative humor, anxiety, and psychological distress), whereas the use of reappraisal is strongly associated with better outcomes (e.g., emotional self-efficacy, benefit finding, and posttraumatic growth), referring to the notion that cognitive strategies are often more adaptative (Sears et al., 2003; Nakatani et al., 2014; Brandão et al., 2017b; Guimond et al., 2019). At the interpersonal level, the limited number of studies suggests that the vast majority of cancer patients benefits from socially sharing their emotions (e.g., Cantisano et al., 2013). For breast cancer in particular, a higher level of sharing avoidance was correlated with more psychological distress and intrusive thoughts (Boinon et al., 2014). Ultimately, both levels of emotion regulation influence the way women diagnosed with breast cancer perceive their current situation, playing an important role in their perception of wellbeing and quality of life. Cella (1994) defends that the quality of life of patients diagnosed with chronic diseases involves two components: multidimensionality and subjectivity. The first component refers to the multiple dimensions of wellbeing that constitute quality of life, specifically physical wellbeing (e.g., symptoms and side effects of treatment), functional wellbeing (e.g., physical capacity and mobility), emotional wellbeing (e.g., negative feelings and concerns) and social wellbeing (e.g., close relationships and social support). For breast cancer in particular, there is another dimension associated to the disease’s specific concerns (e.g., feelings of femininity and swelling of the arms). The second component is related to the notion that quality of life is a subjective construct that can only be evaluated from the patient’s perspective, through self-reporting. As such, the way breast cancer patients perceive their lives, illness, and treatments determine the way they perceive their quality of life (Cella, 1994; Brady et al., 1997). Recent studies show that patients that use adaptative intrapersonal strategies, such as cognitive reappraisal, perceive their quality of life to be better in comparison with when they use strategies which are typically considered more dysfunctional, such as emotional suppression (Brandão et al., 2017b; Lu et al., 2018). The same pattern has been observed for interpersonal regulation, with the sharing avoidance strategy having a negative impact on quality of life (Lai et al., 2017). Additionally, research in other relevant areas further supports this notion. For instance, a couple-based approach to dealing with cancer and related unpleasant experiences—characterized by open and constructive communication (Traa et al., 2015) —has been shown to improve multiple dimensions of quality of life, such as the psychological, physical, and relationship dimensions, the latter being regarded as a component of social and family wellbeing (e.g., Regan et al., 2012; Badr and Krebs, 2013; Traa et al., 2015).

Few studies, however, have compared intrapersonal and interpersonal emotion regulation processes, as well as antecedent-focused and response-focused strategies; to the best of our knowledge, none have done so in the field of breast cancer. Little is known about which level of regulation and which type of strategy have the most beneficial impact on breast cancer patients’ perceived quality of life, especially since the relation between emotional sharing and breast cancer is still in need of further research. To investigate the association between intrapersonal and interpersonal emotion regulation strategies and perceived quality of life, a correlational and cross-sectional study was conducted among women with breast cancer. In particular, the present study contributes to this area by exploring the effects of two levels of emotion regulation (intrapersonal and interpersonal), as well as two types of strategies (antecedent-focused and response-focused) on quality of life in women previously diagnosed with breast cancer. Recollection will be used as a strategy to assess these constructs during the active phase of the disease, including the diagnosis and treatment phases. To allow for a more direct comparison, two well-researched strategies were selected, based on their primary focus during the regulation process for each regulatory level. For intrapersonal level, cognitive reappraisal (antecedent-focused) and emotional suppression (response-focused) were selected, while cognitive sharing (antecedent-focused) and socio-affective sharing (response-focused) were considered for the interpersonal level. Given the severe physical, psychological, and social repercussions of breast cancer, we will focus on decreasing the negative effects of unpleasant emotions. Furthermore, as cancer is a broad term that covers several types of this disease, each with different types of symptoms, health consequences, and lethality, we chose to focus on breast cancer as we were interested in exploring the impact of regulatory processes on patients facing a situation where the underlying challenges are very similar. In addition, this type of cancer has one of the highest survival rates, and thus, these unpleasant emotions can linger over a long period of time, making it essential that these women adopt strategies early on to deal with them effectively.

Firstly, we aim to explore which emotion regulation level and which type of regulatory strategy were most implemented by breast cancer patients to regulate their unpleasant emotions. Likewise, we also assess the levels of consistency when using these strategies, that is, whether the same type of strategy was employed consistently in both regulatory levels. Secondly, we aim to assess the influence of emotion regulation on quality of life, by means of the two regulatory levels and the two types of strategies that were previously outlined. Considering that the area of interpersonal regulation is underdeveloped in the oncological field, we also focus on exploring the role of different indicators of social sharing of emotions in the perception of quality of life.

## Materials and Methods

### Sample Determination

To determine the adequate sample size to test the first objective—that is, to explore the regulatory level and type of strategy most implemented to regulate unpleasant emotions—, we first conducted an *a priori* power analysis, which indicated that the required minimum sample size for a two-way repeated measures ANOVA was between 16 and 36 participants, with 95% power to detect medium- to large-sized effects, for an alpha of 0.05. To test the second objective—that is, to compare the influencing role of emotion regulation on patients’ perception of quality of life—, an *a priori* power analysis indicated that the required minimum sample size for multiple regression analyses with five predictors was between 63 and 138 participants, with 95% power to detect medium- to large-sized effects, for an alpha of 0.05 (G-Power; Faul et al., 2007). At a later stage, during the process of data analyses, the number of predictors decreased from five to two and, as such, the statistical power of this analysis increased. The required minimum sample size for multiple regression analyses with two predictors ranged between 48 and 107 participants, with 95% power to detect medium- to large-sized effects, for an alpha of 0.05. Therefore, we determined that the minimum sample size would be 48.

The inclusion criteria applied in order to select the sample were that all participants had to have been diagnosed with breast cancer but had already completed all required treatments (except for hormone therapy) during their participation in the study. In addition, they had to be 18years old or older and fluent in Portuguese. Women who were at the stage of diagnosis, undergoing treatment, or in an active recurrence stage were considered ineligible to participate to avoid adding an additional factor of emotional vulnerability, since these situations represent acute stages of the disease.

### Participants

Sixty-eight women previously diagnosed with breast cancer participated in this study, with ages ranging from 34 to 81years old (*M*=63.49, *SD*=11.58). Participants were recruited with the collaboration of two Portuguese breast cancer support associations that were able to accommodate our request to collect data in their headquarters. Hence, the study comprises a convenience sample (non-probabilistic method).

About 57.4% of the respondents were married, and 57.6% were retired. Regarding educational levels, 38.1% completed school up to the 9th grade, 23.9% graduated from high school, and 25.4% earned an academic degree. On average, participants had been diagnosed for 118months (*SD*=89.19) and completed their treatments 90months (*SD*=90.49) prior to the study. Of the total, 89.7% of the cases were primary breast cancer cases. All participants underwent surgery, of which 66.2% were mastectomies and 33.8% were lumpectomies. Additionally, they also underwent radiation therapy (70.6%), chemotherapy (58.8%), and hormone therapy (55.9%). The vast majority did not have breast reconstruction surgery (78.5%). About 76.5% had no psychological follow-up, and 77.9% did not participate in support group sessions. Only 10 participants were still undergoing hormone therapy at the time they participated in the study. A detailed description of the sample is presented in Table 1.

**Table 1:** Sociodemographic and clinical characteristics of the sample.

	*n*	%	*M*	*SD*
Age	65	–	63.49	11.58
Marital status	68	–	–	–
Single	2	2.94	–	–
Married	39	57.35	–	–
Civil union	4	5.88	–	–
Divorced/separated	10	14.71	–	–
Widow	13	19.12	–	–
Education	67	–	–	–
4th grade	16	23.88	–	–
9th grade	10	14.93	–	–
High school	16	23.88	–	–
Undergraduate	15	22.39	–	–
Master’s degree	2	2.99	–	–
Other	8	11.94	–	-
Employment situation	66	–	–	–
Active/working	17	25.76	–	–
Unemployed	3	4.55	–	–
Sick leave	3	4.55	–	–
Retired	38	57.58	–	–
Other	5	7.58	–	–
Time since diagnosis (in months)	67	–	117.52	89.19
Type of cancer	68	–	–	–
Original/primary cancer	61	89.71	–	–
Recurrence	7	10.29	–	–
Time since last treatment (in months)	61	–	90.07	90.49
Treatments (more than one possibility)	68	–	–	–
Surgery	68	100.00	–	–
Radiation therapy	48	70.59	–	–
Chemotherapy	40	58.82	–	–
Hormone therapy	38	55.88	–	–
Other	30	44.11	–	–
Still undergoing hormone therapy	68	–	–	–
Yes	10	14.71	–	–
No	58	85.29	–	–
Type of surgery	68	–	–	–
Mastectomy	45	66.18	–	–
Lumpectomy	23	33.82	–	–
Breast reconstruction surgery	65	–	–	–
Yes	14	21.54	–	–
No	51	78.46	–	-
Time since reconstruction (in months)	9	–	134.67	71.19
Psychological follow-up	68	–	–	–
Yes	16	23.53	–	–
No	52	76.47	–	–
Participation in groups and/or associations	68	–	–	–
Yes	15	22.06	–	–
No	53	77.94	–	–

### Measures

Data were collected through a self-report paper questionnaire divided into three main sections. As participants had already finished their cycle of treatments at the time of the study, we used the recall technique to collect our data. This technique is commonly used (see Rimé, 2009; Brans et al., 2013) to assess the emotional sharing experience after the end of a negative episode. Accordingly, all items were adapted to be written in the past tense to redirect participants’ answers toward the active phase of the disease. In the first section, participants were asked to recall an experience during their diagnosis or treatment phase in which they felt a strong negative emotion and then completed the emotional experience, intrapersonal regulation, and social sharing of emotions measures. For this section, the instructions given to the participants were to *“try to remember a specific experience related to your breast cancer diagnosis or treatment in which you felt a strong negative emotion. It is not important what kind of emotion you felt (for example, anger or sadness). What is important is that you think of a specific experience, and that the associated negative emotion was intense. Please answer the first part of the questionnaire based on that particular experience.”* In the second section, respondents assessed their quality of life based on the time they were undergoing breast cancer-related treatment. For this section, the instructions were to *“focus on your overall experience with breast cancer during the time you were undergoing treatment. Please answer this second part of the questionnaire based on that experience in general.”* The third section included questions about their sociodemographic and clinical information.

#### Emotional Experience

The importance of the emotional experience was measured with one item (i.e., “To what extent do you believe the emotional experience was important to you?”), rated on a seven-point scale, ranging from 1 (not very important) to 7 (extremely important). The emotional response was measured with the Self-Assessment Manikin (SAM) by Bradley and Lang (1994). SAM is a non-verbal, pictorial (figure-based) scale, with three items to assess the valence, arousal, and dominance associated with the reaction to an emotional experience. Previous research suggests that participants may experience difficulty understanding how to rate their emotional experience (particularly in the dominance dimension) based on the pictorial response scale (e.g., Korovina et al., 2019). To address this difficulty, we added a label for the lowest and highest point of each dimension. Responses are rated on a nine-point scale, ranging from a frowning figure (1—negative) to a smiling one (9—positive) in the valence dimension, a sleepy figure (1—relaxed) to a wide-eyed one (9—aroused) in the arousal dimension, and a small figure (1—without control) to a big one (9—with maximum control) in the dominance dimension. Overall, higher values indicate positive valence, higher levels of arousal, and greater control over the emotional situation. The original scale revealed good psychometric qualities for valence and arousal. The correlation coefficient between SAM and another equivalent measure was 0.97 for valence and 0.94 for arousal. For dominance, the correlation was non-significant at 0.23 (Bradley and Lang, 1994). However, recent studies have shown a higher correlation coefficient for this dimension (see Bynion and Feldner, 2017).

#### Intrapersonal Emotion Regulation

Intrapersonal regulation was measured using the Portuguese version of the Emotion Regulation Questionnaire (ERQ), adapted to the oncological context by Brandão et al. (2017b), originally by Gross and John (2003). ERQ is a self-report questionnaire with 10 items to assess two main regulatory strategies: cognitive reappraisal with six items (e.g., “I have controlled my emotions by changing the way I think about the situation I’m in”) and emotional suppression with four items (e.g., “I have controlled my emotions by not expressing them”). Responses are rated on a seven-point scale, ranging from 1 (strongly disagree) to 7 (strongly agree). Higher values indicate a greater use of the regulatory strategy. In the original scale, cognitive reappraisal and emotional suppression revealed a good internal consistency with a Cronbach α value of 0.79 and 0.73, respectively (Gross and John, 2003). In the Portuguese scale validated for the oncological context, both strategies revealed an equally good internal consistency with a Cronbach α value of 0.82 for reappraisal and 0.72 for suppression (Brandão et al., 2017b). In this study, internal consistency analyses also revealed a good Cronbach α value of 0.81 for reappraisal and an acceptable Cronbach α value of 0.76 for suppression.

#### Social Sharing of Emotions

To measure social sharing of emotions, six items were selected from key studies in the area (e.g., Nils and Rimé, 2012; Brans et al., 2013; Cantisano et al., 2013; Duprez et al., 2015) to assess the most relevant dimensions of this regulatory process, namely: need to share, sharing occurrence, and sharing benefits. The need to share with someone was evaluated with one item (i.e., “Did you feel the need to share the emotion related to your experience?”). The sharing benefits dimension included four items, namely, two items to assess the general perceived benefits (e.g., “Do you believe sharing was beneficial for you?”), one item to measure the socio-affective sharing mode (i.e., “Do you believe the person you shared your experience with tried to support you?”), as a response-focused strategy, and one item to evaluate the cognitive sharing mode (i.e., “Did the person you shared your experience with encourage you to look at the situation from a different perspective?”), as an antecedent-response strategy. Responses were rated on a seven-point scale, ranging from 1 (not at all) to 7 (very much). The remaining item assessed the sharing occurrence (i.e., “At the time, did you talk to someone about that experience and/or emotion?”). Responses were rated on a seven-point scale, ranging from 1 (I did not share) to 7 (I shared a lot). In all items, higher values indicate greater social sharing indicators. The structural validity of the social sharing of emotions measure was tested with R. The three previous dimensions were considered, that is, need to share, sharing occurrence, and sharing benefits. The incremental indexes reveal an adequate model fit, CFI=0.97, TLI=0.86. TLI and CFI values are adequate for small samples sizes (Barrett, 2007). However, we did not achieve a good fit for absolute indexes, RMSEA=0.18 *χ*^2^(2,68)=6.47 *p*=0.039. This result should be regarded with caution as the sample size is not adequate for this statistical procedure.

#### Breast Cancer-Related Quality of Life

Quality of life was measured using the Portuguese version of Functional Assessment of Cancer Therapy—Breast (FACT-B) by Brady et al. (1997), translated and provided by FACIT. FACT-B is a self-report questionnaire with 37 items to assess quality of life in breast cancer patients from a multidimensional perspective. This measure combines the Functional Assessment of Cancer Therapy—General by Cella et al. (1993) with 10 items on specific breast cancer-related problems. Items were grouped into five subscales: physical wellbeing, with seven items (e.g., “I lacked energy”); social and family wellbeing, with seven items (e.g., “I felt close to my friends”); emotional wellbeing, with six items (e.g., “I felt sad”); functional wellbeing, with seven items (e.g., “I was able to work [including work at home]”); and additional concerns associated with breast cancer, with 10 items (e.g., “I was short of breath”). Responses were rated on a five-point scale, ranging from 0 (not at all) to 4 (very much). Twenty items had to be reverse-coded so that higher values indicated a better breast cancer-related quality of life. In the original scale, internal consistency analyses revealed acceptable to good Cronbach *α* values, with 0.81 for physical wellbeing, 0.90 for social and family wellbeing, 0.67 for emotional wellbeing, 0.87 for functional wellbeing, and 0.60 for additional concerns, with a good Cronbach *α* value of 0.89 for the overall FACT-B scale (Cella et al., 1993). In this study, internal consistency analyses revealed equally good Cronbach *α* values, with 0.86 for physical wellbeing, 0.81 for social and family wellbeing, 0.79 for emotional wellbeing, 0.85 for functional wellbeing, and 0.80 for additional concerns. The overall scale revealed an acceptable Cronbach *α* value of 0.70.

### Procedure

This correlational and cross-sectional study was approved by Iscte’s Ethics Committee and the president of each Portuguese breast cancer support association that collaborated with the study. Data were collected from March to May 2018. Participants were invited to take part in a study about emotion regulation and quality of life in breast cancer. Each participant read an informed consent form, describing the purpose of the study, explaining the conditions of their participation, and safeguarding the anonymity and confidentiality of their responses. They were also informed that their participation was voluntary, and they were free to withdraw at any moment (data and/or participation). After giving their consent, the paper-based questionnaire was completed individually in a quiet and private place at the associations’ headquarters, in a single 20min session. A researcher was present at all times, for when participants felt the need to clarify any point of the questionnaire. The questions raised were of an occasional and idiosyncratic nature. At the end, participants were debriefed and were given more information regarding the research topic. There was no compensation for their participation, monetary, or otherwise. Given the sensitive topic, participants were encouraged to contact the researchers if they needed psychological support during or after their participation.

## Results

Statistical analyses were performed using SPSS software, version 27, with a 95% confidence interval. Descriptive analysis demonstrated that recalled emotional experiences were perceived as important for the participants (*M*=5.52, *SD*=1.59). The emotional response was neutral (*M*=4.60, *SD*=2.32), and participants reported feeling moderately activated by the experience (*M*=5.25, *SD*=2.34), with some control over it (*M*=5.93, *SD*=2.68). The results are summarized in Table 2.

**Table 2:** Descriptive statistics for the emotional variables and quality of life.

Variables	Range	*M*	*SD*	Correlations (Pearson *r*)															
				1	2	3	4	5	6	7	8	9	10	11	12	13	14	15	16
**Emotional experience**																			
1.Importance	1–7	5.52	1.59	–															
2.Valence	1–9	4.60	2.32	−0.35^**^	–														
3.Arousal	1–9	5.25	2.34	0.13	−0.35^**^	–													
4.Dominance	1–9	5.93	2.68	−0.38^**^	0.56^***^	−0.38^**^	–												
**Intrapersonal regulation**																			
5.Cognitive reappraisal	1–7	5.08	1.30	0.29^*^	−0.04	0.12	−0.12	–											
6.Emotional suppression	1–7	3.94	1.64	−0.25^*^	0.18	0.11	−0.04	0.08	–										
**Interpersonal regulation**																			
7.Need to share	1–7	4.60	1.94	0.07	−0.00	0.26^*^	−0.17	0.36^**^	0.58^***^	–									
8.Sharing occurrence	1–7	4.82	1.80	−0.24^+^	0.22^+^	0.07	0.11	0.23^+^	0.86^***^	0.69^***^	–								
9.Sharing benefits	1–7	5.25	1.72	0.08	0.05	0.18	−0.21^+^	0.78^***^	0.34^**^	0.48^***^	0.43^***^	–							
10.Cognitive sharing mode	1–7	5.16	2.11	0.20	−0.06	0.13	−0.14	0.89^***^	0.19	0.31^*^	0.32^**^	0.82^***^	–						
11.Socio-affective sharing mode	1–7	5.64	1.87	0.08	0.00	0.13	−0.19	0.72^***^	0.15	0.23^+^	0.23^+^	0.89^***^	0.81^***^	–					
**Quality of life**																			
12.Physical wellbeing	0–4	2.27	1.05	−0.34^**^	0.47^***^	−0.45^***^	0.44^***^	−0.17	−0.03	−0.30^*^	−0.04	−0.13	−0.20	−0.12	–				
13.Social and family wellbeing	0–4	2.96	0.80	0.06	0.11	0.08	0.10	0.39^**^	0.03	0.23^+^	0.16	0.41^**^	0.29^*^	0.30^*^	−0.04	–			
14.Emotional wellbeing	0–4	2.64	0.82	−0.33^**^	0.37^**^	−0.46^***^	0.41^**^	−0.13	−0.03	−0.36^**^	−0.01	−0.08	−0.08	−0.02	0.51^***^	−0.05	–		
15.Functional wellbeing	0–4	2.41	0.81	−0.38^**^	0.26^*^	−0.40^**^	0.35^**^	0.07	0.09	−0.02	0.25^*^	0.18	0.05	0.15	0.38^**^	0.23^+^	0.48^***^	–	
16.Additional concerns	0–4	2.39	0.76	−0.32^**^	0.29^*^	−0.37^**^	0.32^**^	−0.11	0.06	−0.23^+^	0.09	−0.09	−0.06	−0.07	0.59^***^	0.01	0.61^***^	0.52^***^	–
17.Quality of life	0–20	12.66	2.89	−0.39^**^	−0.46^***^	−0.49^***^	0.49^***^	−0.00	0.03	−0.21^+^	0.12	0.07	−0.02	0.06	0.76^***^	0.32^**^	0.75^***^	0.76^***^	0.80^***^

+ *p*<0.085;

* *p*<0.05;

** *p*<0.01;

*** *p*<0.001.

Regarding intrapersonal emotion regulation, participants used cognitive reappraisal (*M*=5.08, *SD*=1.30) to deal with the emotional burden more than emotional suppression (*M*=3.94, *SD*=1.64). As for interpersonal regulation, participants reported feeling a moderate need to share their experiences (*M*=4.60, *SD*=1.94) and actively shared them with other people (*M*=4.82, *SD*=1.80). The sharing benefits indicator revealed a higher score (*M*=5.25, *SD*=1.72), suggesting that participants found the sharing process to be a positive and beneficial regulatory process. Socio-affective sharing (*M*=5.64, *SD*=1.87) was used more frequently than cognitive sharing (*M*=5.16, *SD*=2.11). Strong and positive correlations were found between reappraisal and sharing benefits, *r*=0.78, *p*<0.001, reappraisal and cognitive sharing, *r*=0.89, *p*<0.001, and reappraisal and socio-affective sharing, *r*=0.72, *p*<0.001; between suppression and need to share, *r*=0.58, *p*<0.001, and suppression and sharing occurrence, *r*=0.86, *p*<0.001. This means that the more patients cognitively reformulated the unpleasant emotional episode, the greater the benefits that arose from emotional sharing; the more they suppressed their feelings, the more they felt the need to share and actively did so.

Overall, participants perceived their quality of life to be average during the time they were undergoing breast cancer treatments (*M*=12.66, *SD*=2.89). Social and family wellbeing (*M*=2.96, *SD*=0.80), as well as emotional wellbeing (*M*=2.64, *SD*=0.82), were perceived to be better than physical wellbeing (*M*=2.27, *SD*=1.05), functional wellbeing (*M*=2.41, *SD*=0.81), and additional concerns specific to breast cancer (*M*=2.39, *SD*=0.76). Significant and positive associations were only found between social and family wellbeing and intrapersonal (reappraisal), *r*=0.39, *p*<0.010, and social and family wellbeing and sharing benefits, *r*=0.41, *p*<0.010, cognitive sharing, *r*=0.29, *p*<0.050, and socio-affective sharing, *r*=0.30, *p*<0.050.

### Comparisons Between Regulatory Levels and Strategies

The first objective of the study was to determine the most implemented regulatory level and type of strategy during the active phase of the disease and analyze the consistency level of strategy use for each regulatory level. A two-way repeated measures ANOVA was conducted to examine the effects of the regulatory level (intrapersonal vs. interpersonal) and type of strategy (antecedent-focused vs. response-focused) on emotion regulation. Both factors were within-subject. Data are summarized in Table 3.

**Table 3:** Means, standard deviations, and analyses of variance in regulatory levels and types of strategies.

Measure	Intrapersonal		Interpersonal	
	*M*	*SD*	*M*	*SD*
Antecedent-focused	5.08	1.30	5.16	2.11
Response-focused	3.94	1.64	5.64	1.87
Source	*F*_(1,66)_		*η* ^2^	
Regulatory level	12.49^**^		0.16	
Type of strategy	5.34^*^		0.08	
Interaction	34.15^**^		0.34	

* *p<* 0.05;

** *p<* 0.001.

The results yielded a significant main effect of regulatory level, *F*(1,66)=12.49, *p*=0.001, $\eta_{p}^{2}$ =0.16. Scheffe post-hoc comparisons indicated that participants regulated their negative emotions within their social interactions more than they did individually. Results also revealed a significant main effect of type of strategy, *F*(1,66)=5.34, *p*=0.024, $\eta_{p}^{2}$ =0.08. Post-hoc tests using Scheffe correction demonstrated that participants preferred using more antecedent-focused strategies to cognitively reformulate the emotional experience than response-focused strategies. There was a significant interaction effect between regulatory level and type of strategy, *F*(1,66)=34.15, *p*<0.001, $\eta_{p}^{2}$ =0.34. The degree of implementation of antecedent-focused strategies was relatively constant across regulatory levels, suggesting that participants tended to implement this type of strategy, such as reappraisal and cognitive sharing, regardless of the regulatory level they were using at the time. The opposite effect can be observed for response-focused strategies, namely, suppression and socio-affective sharing. When women had a stronger preference for implementing the suppression strategy on an intrapersonal level to cope with their negative emotions, they would not engage in socio-affective sharing with other people and vice-versa.

### Effects of Emotion Regulation on Breast Cancer-Related Quality of Life

The second aim was to explore the relationship between emotion regulation and breast cancer-related quality of life. To perform these analyses, we calculated four new indexes: antecedent-focused strategy, response-focused strategy, intrapersonal regulatory level, and interpersonal regulatory level. The antecedent-focused index was obtained by averaging the cognitive reappraisal score (intrapersonal regulatory strategy) with the cognitive sharing item (interpersonal regulatory strategy), *r*=0.26, *p*=0.031; the response-focused index was calculated by averaging the reversed emotional suppression score (intrapersonal strategy) with the socio-affective sharing item (interpersonal strategy), *r*=0.32, *p*=0.009. Similarly, the intrapersonal level index was obtained *via* the average of the 10 items of the ERQ, *α*=0.75; the interpersonal level index was obtained *via* the average of the cognitive mode and socio-affective sharing items of social sharing of emotions, *r*=0.81, *p*<0.001.

A first set of multiple linear regressions was conducted to assess the association between the five dimensions of quality of life (as criterion variables) and antecedent-focused and response-focused emotion regulation strategies (as predictors). Age and cancer recurrence (i.e., first time having cancer vs. recurrence) were controlled in these analyses. We used bootstrapping, as it has a relatively higher power for detecting smaller effects. Results are summarized in Table 4. According to Cohen (1992), a r-square value of 0.12 or below indicates a low effect size, values between 0.13 to 0.25 indicate a medium effect size, and values of 0.26 or above indicate high effect size. Data show that social and family wellbeing were positively associated with the use of antecedent-focused emotion regulation strategies, *b*=0.25, *t*(60)=4.05, *p*<0.001, *R*^2^=0.19, but not with response-focused ones, *b*=−0.01, *t*(60)=−0.21, *p*=0.836, *R*^2^=0.00. None of the remaining associations were statistically significant.

**Table 4:** Regression analyses for quality of life measures by types of strategy and regulatory levels.

	*b*	*SE b*	*t*	*p*
**Physical wellbeing**				
Antecedent-focused strategies	−0.10	0.09	−1.03	0.309
Response-focused strategies	−0.03	0.09	−0.38	0.709
Age	0.01	0.01	1.39	0.169
Cancer recurrence	0.31	0.44	0.70	0.487
*R*^2^ = 0.08; *R*^2^_adjusted_ = 0.02; *F*_(4,60)_ =1.26; *p* =0.298, Effect size =0.44				
Intrapersonal level	−0.01	0.12	−0.10	0.919
Intrapersonal level	−0.04	0.07	−0.53	0.600
Age	0.02	0.01	1.45	0.153
Cancer recurrence	0.24	0.45	0.54	0.590
*R*^2^ = 0.07; *R*^2^_adjusted_ = 0.00; *F*_(4,60)_ =0.98; *p* =0.426, Effect size=0.38				
**Social and family wellbeing**				
Antecedent-focused strategies	0.25	0.06	4.05	0.000
Response-focused strategies	−0.01	0.06	−0.21	0.836
Age	0.02	0.01	2.90	0.005
Cancer recurrence	0.87	0.29	2.98	0.004
*R*^2^ = 0.31; *R*^2^_adjusted_ = 0.26*; F*_(4,60)_ =6.71; *p* <0.000, Effect size=0.99				
Intrapersonal level	0.30	0.08	3.71	0.000
Interpersonal level	0.11	0.05	2.17	0.034
Age	0.02	0.01	2.66	0.010
Cancer recurrence	0.92	0.30	3.08	0.003
*R*^2^ = 0.30; *R*^2^_adjusted_ = 0.25; *F*_(4,60)_ =6.41; *p* <0.000, Effect size=0.99				
**Emotional wellbeing**				
Antecedent-focused strategies	−0.11	0.07	−1.52	0.134
Response-focused strategies	0.04	0.07	0.57	0.572
Age	0.02	0.01	1.65	0.103
Cancer recurrence	0.16	0.34	0.47	0.640
*R*^2^ = 0.10; *R*^2^_adjusted_ = 0.03; *F*_(4,60)_ =1.57; *p* =0.194, Effect size=0.55				
Intrapersonal level	−0.15	0.10	−1.60	0.116
Intrapersonal level	−0.03	0.06	−0.49	0.625
Age	0.02	0.01	1.72	0.091
Cancer recurrence	0.20	0.35	0.56	0.577
*R*^2^ = 0.10; *R*^2^_adjusted_ = 0.04; *F*_(4,60)_ =1.60; *p* =0.186, Effect size=0.56				
**Functional wellbeing**				
Antecedent-focused strategies	0.13	0.07	1.18	0.858
Response-focused strategies	0.08	0.07	1.13	0.263
Age	0.02	0.01	1.93	0.058
Cancer recurrence	0.34	0.33	1.02	0.313
*R^2^ =* 0.08; *R^2^*_adjusted_ = 0.02; *F*_(4,60)_ =1.24; *p* =0.302, Effect size=0.44				
Intrapersonal level	0.10	0.09	1.12	0.266
Intrapersonal level	0.12	0.05	2.25	0.028
Age	0.02	0.01	1.88	0.065
Cancer recurrence	0.41	0.34	1.23	0.225
*R*^2^ = 0.13; *R*^2^_adjusted_ = 0.08; *F*_(4,60)_ =2.30; *p* =0.046, Effect size=0.69				
**Breast cancer-related additional concerns**				
Antecedent-focused strategies	−0.08	0.07	−1.15	0.254
Response-focused strategies	0.07	0.07	1.07	0.288
Age	0.02	0.01	2.27	0.027
Cancer recurrence	0.35	0.32	1.11	0.273
*R*^2^ = 0.15; *R*^2^_adjusted_ = 0.09; *F*_(4,60)_ =2.67; *p* =0.041, Effect size=0.77				
Intrapersonal level	−0.10	0.09	−1.18	0.244
Intrapersonal level	0.02	0.05	0.42	0.674
Age	0.02	0.01	2.29	0.026
Cancer recurrence	0.36	0.32	1.10	0.277
*R*^2^ = 0.15; *R*^2^_adjusted_ = 0.09; *F*_(4,60)_ =2.60; *p* =0.050, Effect size=0.77				

Similarly, a second set of multiple linear regressions with bootstrapping was conducted to assess the association between the five dimensions of quality of life (as criterion variables) and the intrapersonal and interpersonal regulatory levels (as predictors). Again, age and cancer recurrence were controlled in the analyses. Social and family wellbeing was positively predicted by both regulatory levels. However, intrapersonal regulation was more strongly associated with social and family wellbeing than interpersonal regulation, *b*=0.30, *t*(60)=3.71, *p*<0.001, *R*^2^=0.16, *b*=0.11, *t*(60)=2.17, *p*=0.034, *R*^2^=0.05, respectively. Interpersonal regulation positively predicted functional wellbeing, *b*=0.12, *t*(60)=2.25, *p*=0.028, *R*^2^=0.07. The remaining associations were not statistically significant.

A post-hoc statistical power analysis was performed using G-Power (Erdfelder et al., 1996) for the estimation of the sample size. The effect sizes (ES) are reported in Table 4 and vary between 0.38 and 0.99, thus being considered to be medium to large using Cohen's (1988) criteria.

Lastly, to further explore the association between interpersonal regulation and quality of life, we performed a linear regression with bootstrapping between the need to share, sharing occurrence, and sharing benefits and the total score of breast cancer-related quality of life, controlling for age and cancer recurrence. The regression model was significant, *R*^2^=0.27, *F*(5,58)=4.32, *p*=0.002. The results revealed a negative and significant association between the need to share and quality of life, *b*=−0.83, *t*(58)=−3.56, *p*=0.001, *R*^2^=0.14, and a positive and significant association between the occurrence and quality of life, *b*=0.64, *t*(58)=2.50, *p*=0.015, *R*^2^=0.08. The association between sharing benefits and quality of life was not significant, *b*=0.27, *t*(58)=1.23, *p*=0.223. Age had a significant impact on quality of life, *b*=0.27, *t*(58)=2.22, *p*=0.030, but not cancer recurrence *b*=0.24, *t*(58)=1.97, *p*=0.054. The total effect size resulting from a post-hoc statistical power analysis was 0.98, which is considered a large effect (Cohen, 1988).

## Discussion

Breast cancer is an invasive disease that has profound implications for the wellbeing and quality of life of the affected population. Women diagnosed with this disease repeatedly have to learn how to adapt and effectively deal with its negative impact, relying on different regulation processes. This study focuses on the established relationship between emotion regulation and quality of life in breast cancer patients.

The first objective was to compare the implementation of intrapersonal and interpersonal regulatory levels and antecedent-focused and response-focused types of strategies in the breast cancer field. The results yielded significant emotion regulation patterns. For instance, participants actively chose to engage more in social sharing episodes, suggesting that, when possible, they preferred seeking the help of others to assist them in regulating their emotions, rather than do it in isolation. In crisis situations, people often face a discrepancy between the difficulty of the problem and the resources they have at their disposal to cope with it (Caplan, 1964). In fact, Hobfoll’s (1989) seminal theory of conservation of resources posits that individuals strive to protect and build resources and that what threatens people in such situations is the potential or actual loss of those resources. Hence, if breast cancer patients do not believe they have sufficient resources to deal with the emotional burden and vulnerabilities associated with the disease, they might find intrapersonal regulation to be particularly difficult to implement. Interpersonal regulation works as an extra source of support and capabilities that go beyond the personal regulatory skills, potentially helping them overcome such obstacles (e.g., Levy-Gigi and Shamay-Tsoory, 2017). Likewise, the greater need to share might be associated with how patients perceive their emotion regulation self-efficacy. Participants also used antecedent-focused strategies more than response-focused strategies. Since cognitive strategies tend to be the focus of regulatory efforts and are generally more effective (Gross, 2015), resulting in better emotional outcomes (Webb et al., 2012), it is possible that women seek to implement them more often as a way to significantly reduce the levels of arousal and unpleasant impact in both the short and the long term. Furthermore, the use of these strategies was consistent across regulatory levels. Given that reappraisal and cognitive sharing are based on the same premise, that is, to effectively deal with an emotional event, one needs to engage in cognitive reformulation processes to alter the experience’s subjective meaning (Brans et al., 2013; Gross, 2015), these strategies can be considered equivalent, operating at different regulatory levels. These results suggest that it is likely for patients to implement the same type of strategy, as long as it is consistent with the established regulation goal, with the regulatory level representing a means through which the goal is achieved. However, the opposite pattern emerged for response-focused strategies, possibly because they are based on different theoretical assumptions. Suppression actively seeks not to express what is being felt (Gross, 2015), whereas socio-affective sharing implies that the person freely displays their feelings to others (Brans et al., 2013). Thus, when one is being employed, the other is automatically rejected as a potential strategy to use.

The second objective was to explore the relationship between emotion regulation and quality of life in breast cancer patients, during the active phase of the disease. Aligned with previous studies (e.g., Brandão et al., 2017b; Lai et al., 2017; Lu et al., 2018), our results show that, overall, emotion regulation significantly contributed to explain differences in the perception of social and family wellbeing, functional wellbeing, and breast cancer-related quality of life. The greater use of antecedent-focused strategies—known for their cognitive effort in reformulating unpleasant emotional episodes—in particular was associated with a better perception of social and family wellbeing. This result supports the notion that an adaptive regulation, irrespective of the regulatory level, strengthens the social and positive ties between the people involved in the regulatory process (e.g., Nyklíček et al., 2011; Rimé, 2018).

Similarly, when comparing the two regulatory levels being studied, the analyses confirmed that both intrapersonal and interpersonal regulation had a positive effect on social and family wellbeing, thus improving patients’ perceptions of aspects, such as their close relationships and social support. Surprisingly, this effect was stronger for intrapersonal regulation. Given the nature of our study, it is difficult to draw conclusions regarding this result. Another interesting finding is that interpersonal strategies were positively associated with functional wellbeing. When experiencing or recalling breast cancer experiences, there is a risk of strong, unpleasant, and emotion-eliciting episodes, which might encourage patients to actively try to deal with these negative emotions in order to restore a neutral or positive emotional state. It is possible that they feel that, by sharing their unpleasant thoughts and feelings, they can continue to work, function properly (e.g., have better mobility and sleep), and enjoy life. It may also be the case that, by sharing, people receive instrumental social support that enables them to improve their quality of life at this level (House, 1987). Hence, the option to share represents a way to minimize these negative consequences which may arise from the emotional process of adapting to the illness.

On the interpersonal level, specifically, results showed that sharing occurrence positively contributed to patients’ perceived quality of life, whereas sharing benefits had no effect on this perception. Despite the notion that verbalization is not sufficient (Rimé, 2009), this finding indicates that the mere occurrence of sharing is sufficient to promote the perception of quality of life. As such, the social interactions through which women can actively share their feelings and resolve them are the most important factor. Data also yielded a negative relation between the need to share and quality of life. Higher levels of need to share might be counterproductive, especially if patients feel that their urge to talk has not been satisfied, which leads to a lower satisfaction with the sharing event or even with the listeners, resulting in a poorer perception of quality of life. This result is similar to the one found in the study by Boinon et al. (2012), where patients who were not satisfied with their listeners’ reactions during sharing episodes presented more negative emotions.

The results should be interpreted with caution. As a correlational study, causality relations cannot be established between variables. The sample size was small, which also constitutes a limitation of the study. This was due to the limited access to the target population and the time limitations of the participants to respond to the questionnaire. Additionally, most of the women who had access to breast cancer associations had already finished their treatment cycle prior to the study and, therefore, we had to ask them to recall a particular negative experience to collect the data, in congruence with Rimé’s previous work (see Rimé, 2009; Brans et al., 2013). As such, given the retrospective nature of the study, it is possible that participants were at risk of a recall bias, as a significant amount of time had passed since the experience. Besides, the active phase of the disease, which was the focus of the study, is comprised of a series of subphases (e.g., diagnosis and treatment), each with different emotional challenges and/or regulatory needs that were not fully considered in this study. It might be interesting for future studies to assess the effects of these strategies during different subphases, as well as compare the intrapersonal and interpersonal emotion regulation processes during the active and remission phases of breast cancer. Moreover, future studies should also consider testing if the observed relationships can be applied to other types of cancer in a similar way. We suggest, however, some methodological adjustments to enrich the research design, particularly for assessing interpersonal regulation, since the items used in the study do not represent a single measure. Recently after we implemented this study’s protocol, Williams et al. (2018) developed the Interpersonal Regulation Questionnaire, which may be a promising avenue for new research in this area. Likewise, it might be of interest to explore the individual (e.g., personality traits) and social (e.g., social support) factors that have an impact on the efficacy of each regulatory level and type of strategy. Furthermore, only negative emotions were considered in the study, given their persistent nature in this context, but future research should consider the role of positive emotions in each regulatory level and assess their potential as a protection factor against the disease.

Nevertheless, our results constitute an important first step in the comparative study of intrapersonal and interpersonal regulation in the context of breast cancer, as well as the effects of antecedent-focused vs. response-focused strategies. As previously mentioned, studies have focused on the role of interpersonal regulation, and comparative research between intrapersonal and interpersonal regulation is limited in the oncological context, contrary to the vast majority of studies on the role of intrapersonal regulation. This study recognizes the importance of taking into consideration different levels of emotion regulation and types of regulatory strategies when adapting to the disease in order to enhance one’s quality of life. Since interpersonal regulation revealed a positive, beneficial impact, it might be particularly helpful for breast cancer patients to engage in regulatory processes with their most immediate social relationships, as long as the individuals are willing to get involved in the sharing process. Similarly, and in line with recent research (e.g., Hamama-Raz et al., 2016), group intervention, support groups, or even online forums also represent powerful emotion regulation tools, as they provide a safe environment where breast cancer patients can express their feelings, share their thoughts and disease-related concerns, trade useful information, and learn new techniques to better manage their condition. In return, these women feel more empowered and get the support they need to cope with the disease from people that are in similar situations. However, more must be learned about how these groups can foster productive interpersonal regulatory moments. The finding that the vast majority (77.9%) of the participants did not participate in support group sessions highlights the importance of considering with whom interpersonal regulatory efforts are sought. Rimé (2009) states that, for most adults, the main sharing targets are close people with whom they have a deep and intimate relationship (e.g., partners), followed by other close relationships (e.g., family and friends). Thus, it is of importance that patients first develop a relationship of trust and closeness with the members of these groups, in order to perceive them as valid sharing targets. Ultimately, interpersonal regulation has the potential to help breast cancer patients better respond to the disease, helping them lead a more adaptive and positive life.

## Data Availability Statement

The data that support the findings of this study are available from the corresponding author upon reasonable request. The data are not publicly available due to privacy and ethical restrictions.

## Ethics Statement

The study was reviewed and approved by Iscte - Instituto Universitário de Lisboa’s Ethics Committee. The participants provided their written informed consent to participate in the study.

## Author Contributions

RM: conceptualization, investigation, methodology, visualization and data presentation, writing (original and final drafts), and review and editing. CC: conceptualization, methodology, formal analysis, writing (original and final drafts), and review and editing. SL: conceptualization, formal analysis, visualization and data presentation, writing (original and final drafts), and review and editing. All authors contributed to the article and approved the submitted version.

## Funding

This work was supported by Portuguese National Funds through FCT—Fundação para a Ciência e a Tecnologia under the project UIDP/00713/2020.

## Conflict of Interest

The authors declare that the research was conducted in the absence of any commercial or financial relationships that could be construed as a potential conflict of interest.

## Publisher’s Note

All claims expressed in this article are solely those of the authors and do not necessarily represent those of their affiliated organizations, or those of the publisher, the editors and the reviewers. Any product that may be evaluated in this article, or claim that may be made by its manufacturer, is not guaranteed or endorsed by the publisher.
